# Planar Elliptical Inductor Design for Wireless Implantable Medical Devices

**DOI:** 10.3390/bioengineering10020151

**Published:** 2023-01-23

**Authors:** Muhammad Farooq, Bilal Amin, Adnan Elahi, William Wijns, Atif Shahzad

**Affiliations:** 1 Smart Sensors Lab, School of Medicine, University of Galway, H91 TK33 Galway, Ireland; 2 Electrical and Electronic Engineering, University of Galway, H91 TK33 Galway, Ireland; 3 Centre for Systems Modeling and Quantitative Biomedicine, University of Birmingham, Birmingham B15 2TT, UK

**Keywords:** area transformation, circular planar inductor, elliptical inductor, numerical model, narrow implantable locations, planar inductor, wireless implantable medical devices, wireless connectivity

## Abstract

Wireless implantable medical devices (WIMDs) have seen unprecedented progress in the past three decades. WIMDs help clinicians in better-understanding diseases and enhance medical treatment by allowing for remote data collection and delivering tailored patient care. The wireless connectivity range between the external reader and the implanted device is considered one of the key design parameters in WIMD technology. One of the common modes of communication in battery-free WIMDs is inductive coupling, where the power and data between the reader and the implanted device are transmitted via magnetically coupled inductors. The design and shape of these inductors depend on the requirements of the application. Several studies have reported models of standard planar inductors such as circular, square, hexagonal, and octagonal in medical applications. However, for applications, constrained by narrow implantable locations, elliptical planar inductors may perform better than standard-shaped planar inductors. The aim of this study is to develop a numerical model for elliptical inductors. This model allows for the calculation of the inductance of the elliptical planar inductor and its parasitic components, which are key design parameters for the development of WIMDs powered by inductive coupling. An area transformation technique is used to transform and derive elliptical inductor formulas from standard circular inductor formulas. The proposed model is validated for various combinations of the number of turns, trace width, trace separation, and different inner and outer diameters of the elliptical planar inductor. For a thorough experimental validation of the proposed numerical model, more than 75 elliptical planar inductors were fabricated, measured, and compared with the numerical output of the proposed model. The mean error between the measured inductor parameters and numerical estimates using the proposed model is <5%, with a standard deviation of <3.18%. The proposed model provides an accurate analytical method for estimating and optimizing elliptical planar inductor parameters using a combination of current sheet expression and area transformation techniques. An elliptical planar inductor integrated with a sensing element can be used as a wireless implant to monitor the physiological signal from narrow implantation sites.

## 1. Introduction

Wireless implantable medical devices (WIMDs) have gained significant attention due to their suitability for home monitoring and diagnostic surveillance of various devices, including pacemakers, cardiac defibrillators, insulin pumps, and neurostimulators [1,2]. These devices can also enable the monitoring of bone repair and joint stress, which are typically evaluated by modeling [3]. WIMDs have enabled remote patient monitoring and the delivery of personalized care [4,5]. The wireless linkage distance between the WIMD and the external reader device poses a significant challenge while designing and implanting the WIMD inside the human body [6,7]. The wireless linkage range between the implantable device and the reader system is impacted by signal losses due to the interposition of body tissue layers such as skin, fat, muscle, and others [8]. Electrical inductors are commonly used in biomedical implants to provide the wireless power supply or to wirelessly communicate with an external reader for monitoring physiological parameters such as pressure [9,10], blood flow [11], temperature [12], and heart rate [13,14]. In such applications, the planar inductors are printed on printed circuit boards (PCB) and are implanted at the target location inside the body [15]. Numerous studies have demonstrated the use of a planar inductor with a pressure-sensitive capacitive element to develop an LC-based pressure sensor for wearable and implantable applications [16,17,18,19,20].

The planar inductors can be of various shapes, such as circular, octagonal, hexagonal, and square [21]. The selection of any specific inductor shape depends on the application and the implantation site [22]. Square-shaped spirals are most commonly used due to their simple layout [23]. To improve the performance of the spiral inductor, different polygon-shaped inductors have been reported in the literature [24]. Deng et al. [25] reported a pressure sensor made from a capacitor and square-shaped planar inductor for wound monitoring. Chen et al. [13] reported a wireless sensor fabricated using a square-shaped planar inductor to monitor intracranial pressure. Park et al. [10] reported a wireless pressure sensor consisting of a capacitive element and a squared-shaped planar inductor to integrate inside the biodegradable polymer stent for biomedical applications. Chen et al. [26] reported a pressure sensor consisting of a circular planar inductor and capacitor to monitor the intraocular pressure. Symmetrically shaped inductors, such as circular, square, and polygons, have been the primary choice for these sensor designs due to the availability of mathematical models for these shapes of inductors and the needs of the applications.

Various studies have reported the analytical formulas for circular, octagonal, hexagonal, and square-shaped planar inductors [23,27,28,29]. However, these analytical formulas are not valid for semi-symmetrical shaped inductors. The lumped circuit model approach is commonly used to model the spiral inductors and parasitic components. However, the expression to calculate inductance showed some inaccuracies [29]. The exact inductance for the spiral inductor can be calculated using the well-known Maxwell equations [30]. Maha et al. [31] reported a concept of smart cities and integrated sensor systems using displacement sensors, where different topologies of planar inductors were modeled in ANSYS to optimize the sensor response. Iftikhar et al. [32] proposed a method to compute the inductance of a spiral inductor with a 7% error; however, this method was only validated for a circular planar inductor and may not be valid for other symmetrical or semi-symmetrical geometries. Three-dimensional (3D) finite element simulators such as COMSOL Multiphysics [33,34] and ANSYS Maxwell [35] can be used to simulate the inductance for spiral inductors with good accuracy [28]. However, these simulators require long and intense computational power to verify the inductor design [14,23].

In 1962, Grover et al. [36] reported the basic formulas to compute the self-inductance of the current-carrying electrode. The total inductance is computed by adding the nearby electrode’s mutual inductance. This technique has good accuracy but takes longer runtime to compute the inductance. Moreover, this approach is only suitable when the inductor has a whole number of turns [27,37]. In 1974, Greenhouse et al. [27] proposed another approach to computing the inductance of squared-shaped micromachined PCB planar inductors. However, this approach cannot provide an inductance figure directly from design specifications. Crols et al. [38] and Dill et al. [39] reported a simpler formula to compute the inductance of planar inductors. Again, typical errors were found to be around 10–20%, and the analytical model was only validated for a square planar inductor with very few samples. Due to the higher percentage of errors, these expressions were unsuitable for inductor design and optimization [23]. Wheeler et al. [40] reported an expression for computing radio coil inductance; however, the proposed expression was only accurate for circular solenoid coils. Mohan et al. [23] proposed a simple and relatively accurate model for planar spiral inductors. The authors proposed three approximation techniques in [23]. In the first approximation technique, the Wheeler expression was modified, while in the second, an expression was derived from electromagnetic principles by approximating the sides of the spirals as current sheets. In the third approximation, an expression was derived by fitting a mathematical model to a large database of inductors. The average error while computing the inductance of the spiral inductor using these approximated expressions was found to be between 2 and 3% only. The accuracy of the current sheet formula remained between 2 and 3% when the trace separation was smaller than or equal to the trace width. However, the error increases significantly when the ratio between trace separation and trace width becomes larger. The maximum error was found to be 8% when the trace separation was less than or equal to three times trace width.

Andrei et al. [41] reported a technique to model planar inductors through Electromagnetic simulation and by fitting a polynomial expression on the S-parameters of the experimental square planar inductors. This technique is not suitable for inductor modeling as S-parameters vary with the surrounding environment. Laurent et al. [42] reported a comparative study to compute the copper loss of planar inductors at higher frequencies between the 2D and 3D simulators with an error of 5–10% over a frequency range of 6 MHz. However, the authors have not reported the results of the inductance. Claudia et al. [43] reported planar inductor modeling software (CIBSOC) with a user-friendly interface that is simpler to use and takes a short time for inductance computation. This software performs computation using the current sheet expression and can only compute inductance for symmetrical planar inductors.

Most of the analytical models presented in the literature are geometry dependent and only valid for symmetrical planar inductors [23,27,28,29]; therefore, these analytical models cannot be applied directly to semi-symmetrical geometries. The expression reported by Grover et al. [36] is computationally complex and takes a long time to evaluate the inductance; moreover, it was only validated for square geometry. The simpler analytical expressions reported by Wheeler [40], Crols [38], and Dill [39] show typical errors of between 10 and 20%. The percentage error using the Mohan expression also increases if the geometric parameters are not selected within the constraint, such as trace separation (S) ≈ trace width (W) [23]. Finite element simulators such as COMSOL Multiphysics [33,34] and ANSYS Maxwell [35] take a long time and high computational power to simulate the inductor, and the simulation cannot be generalized for different geometries [14,23]. In some of the relevant literature, the analytical models are validated against the simulator; however, the percentage error tends to increase when results are experimentally compared due to the lack of real-life factors in the simulations [23]. The analytical models reported in previous publications have limitations due to geometry, design parameters, higher percentage error, and complex computations [27,37]. Moreover, the current analytical models do not apply to semi-symmetrical geometry, such as elliptical planar inductors which can be beneficial in catheter applications for narrow-site implantation. An elliptical inductor can be integrated with a piezo capacitive sensing element and can be used as an LC wireless sensor to monitor physiological parameters such as respiration, blood pressure, or intraocular pressure [8,16,20,44,45]. Due to the minimized dimension along the major axis of the elliptical inductor, it can be folded into a compact shape and can be delivered to a remote implantation site using a catheter or a similar delivery system. Despite this important use-case of elliptical planar inductors, no analytical model of elliptical inductors has been reported in the literature that computes inductor parameters using simple analytical expressions without the use of finite element simulations, and provides inductor parameters within a small error margin (<5%).

This study aims to develop a numerical model of elliptical planar inductors that is computationally simple and provides inductor parameters that can be verified experimentally. The proposed model will allow for the computation of key inductor parameters such as inductance and other parasitic components. Elliptical planar inductors can be useful in various medical implantable applications where symmetrical shapes cannot be used due to narrow implantation site constraints. This has been achieved using the existing geometrical theorem to transform the design parameters, such as inner diameters (minor and major) and outer diameters (minor and major) of the elliptical planar inductor into the inner and outer diameters of a circular planar inductor. The proposed model was numerically evaluated using MATLAB and was tested for more than 75 inductors while varying the trace width, trace separation, number turn, and major-to-minor axis ratio. In this study, more than 75 elliptical planar inductors were designed, fabricated, and measured. The proposed model was also experimentally evaluated using fabricated elliptical inductors with different design parameters. The results of the proposed model were compared and validated with the experimental results. The error was found to be less than 5% with a standard deviation of 3.18%, which is comparable with the existing literature.

## 2. Materials and Methods

**Figure 1:**
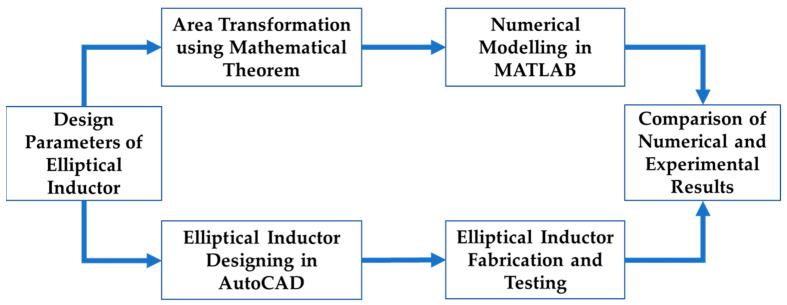
Overall workflow to optimize the design of elliptical inductors.

The elliptical planar inductor shape is novel, and there is no existing expression to calculate the inductance and other parasitic parameters. An ellipse can be represented using an equivalent circular shape using the simple area transformation mathematical theorem. Therefore, using this theorem, all the design parameters (inner and outer major and minor diameters) of the elliptical planar inductor can be transformed into the design parameters (inner and outer diameters) of a circular planar inductor. Figure 1 represents the overall research approach, where it can be seen that the proposed numerical model is validated against the experimental results. 

### 2.1. Planar Inductor Design

As discussed in the introduction, the inductance of the planar inductor depends on the geometrical parameters of the inductor design, which include inner diameter $\left(d_{in}\right)$, outer diameter $\left(d_{out}\right)$, number of turns (N), trace width $\left(W\right),$ and trace separation $\left(S\right).$ The inner or outer diameter of the planar inductor can be calculated from Equation (1) [46].

(1) $$d_{out}=d_{in}+2N\left(S+W\right)+W$$

**Figure 2:**
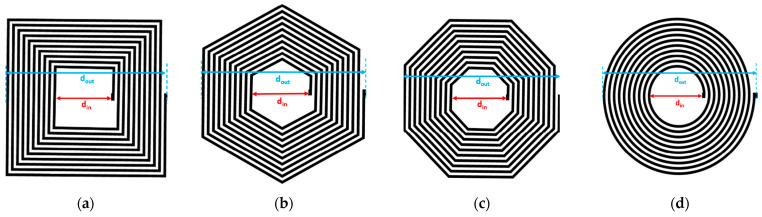
Symmetrical spiral planar inductors (**a**) square (**b**) Hexagonal (**c**) Octagonal (**d**) Circular.

Equation (2) represents the analytical formula to calculate the inductance of symmetrically shaped planar inductors [23]. Equation (2) is derived explicitly for circular, octagonal, hexagonal, and square-shaped planar inductors [23], as shown in Figure 2. In Equation (2), $C_{1}$, $C_{2}$, $C_{3},$ and $C_{4}$ are geometrical constants and their values are given in Table 1 [23].

(2) $$L=\frac{µN^{2}d_{avg}C_{1}}{2}\left(\ln\left(C_{2}/\tau \right)+C_{3}\tau +C_{4}\tau^{2}\right)$$

where, $d_{avg}=\frac{(d_{in}+d_{out)}}{2}$, $\tau =\frac{\left(d_{out\;}–\;d_{in}\right)}{\left(d_{out\;}+d_{in}\right)}$, $C_{1}$, $C_{2}$, $C_{3}$ and $C_{4}$.

Here $d_{avg}$ is the average diameter of the planar inductor and τ represents the fill ratio of the inductor, which is an indicator of how hollow the inductor is; a smaller τ corresponds to a hollower inductor and $d_{out}$ and $d_{in}$ are approximately similar. From Equations (1) and (2), it is evident that the inductance of the planar inductor depends on the inductor’s geometrical parameters. From Figure 2, it can be observed that all the planar designs are symmetrical in shape. The constants listed in Table 1 are specifically computed for symmetrical shapes. If the shape of the inductor becomes asymmetrical or semi-symmetrical, then these constants cannot be used directly. The elliptical planar inductor is a semi-symmetrical shape; therefore, the inductance of the elliptical inductor cannot be directly calculated from Equation (2) in combination with the constants given in Table 1. In the current study, an area transformation concept is used to calculate the inductance of an elliptical planar inductor using a simple mathematical translation technique.

### 2.2. Area Transformation Technique to Model Elliptical Planar Inductor

**Figure 3:**
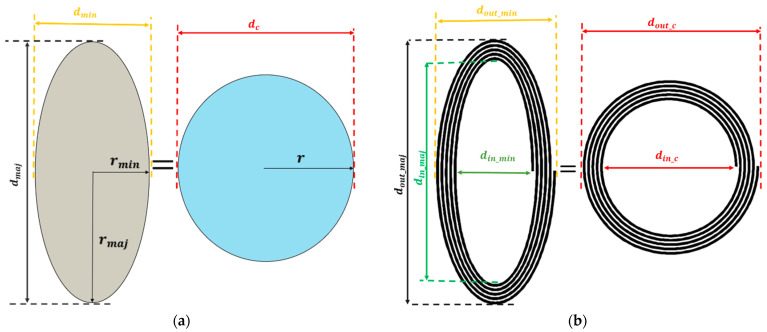
Area transformation technique (**a**) Ellipse area transformation to a circular shape (**b**) Elliptical planar inductor transformation into the circular planar inductor.

In this paper, an area transformation technique is used to transform the area of an elliptical shape into a circular shape [47]. In this approach, the inductance of the elliptical planar inductor ($L_{ellipse}$) is estimated by translating the area of the ellipse ($A_{ellipse})$ into the area of the circle $\left(A_{circle}\right)$ by using mathematical transformation formulas [47]. Using this technique, the design parameters of the elliptical planar inductors are transformed into a circular planar inductor, and all the circular planar inductor formulas can be reused. Figure 3 represents the schematic flow of the transformation of the ellipse area into the area of the circle. Figure 3a shows an ellipse with a minor radius $\left(r_{min}\right)$ and major radius $\left(r_{maj}\right),$ which can be transformed into a circle with a radius $\left(r\right)$ using Equations (3)–(5). Equations (6) and (7) represent the radius (r) and diameter $\left(d_{c}\right)$ of the translated circle.

(3) $$A_{ellipse}=A_{circle}$$

while

(4) $$A_{ellipse}=\pi \;r_{min}r_{maj}$$

(5) $$A_{circle}=\pi \;r^{2}$$

substituting Equations (4) and (5) into Equation (3),

$$\pi \;r^{2}=\pi \;r_{min}r_{maj}$$

(6) $$r=\sqrt{r_{min}r_{maj}}$$

(7) $$d_{c}=2\sqrt{r_{min}r_{maj}}$$

Similarly, the elliptical planar inductor in Figure 3b can also be translated into a circular planar inductor by using Equation (7). Therefore, the inner diameter $\left(d_{in\_c}\right)$ and outer diameters $\left(d_{out\_c}\right)$ of the translated circular planar inductor in terms of elliptical inner diameter $\left(d_{in\_min},d_{in_{maj}}\right)$ and outer diameter$\;(d_{out\_min},d_{out\_maj})$ can be given as follows in Equations (8) and (9):

(8) $$d_{in\_c}=\sqrt{d_{in\_min}d_{in\_maj}}$$

similarly,

(9) $$d_{out\_c}=\sqrt{d_{out\_min}d_{out\_maj}}$$

After transforming an elliptical planar inductor into a circular planar inductor, which is a standard shape of a planar inductor, all formulas of a circular planar inductor can be used to estimate the inductance of an equivalent planar elliptical inductor through Equation (2). The inductance of the elliptical planar inductor is given by Equation (10). Moreover, the parasitic components can also be calculated using the standard formulas of the circular planar inductor.

(10) $$L_{ellipse}=\frac{µN^{2}d_{avg\_ellipse}C_{1}}{2}\left(\ln\left(C_{2}/\tau_{ellipse}\right)+C_{3}\tau_{ellipse}+C_{4}\tau_{ellipse}{}^{2}\right)$$

where, $d_{avg\_ellipse}=\frac{\left(d_{in\_c}+d_{out\_c}\right)}{2}$, $\tau_{ellipse}=\frac{\left(d_{out\_c}–\;d_{in\_c}\right)}{\left(d_{out\_c}+d_{in\_c}\right)}$, $C_{1}$, $C_{2}$, $C_{3},$ and $C_{4}$ are geometry dependent and listed in Table 1.

### 2.3. Parasitic Components

**Figure 4:**
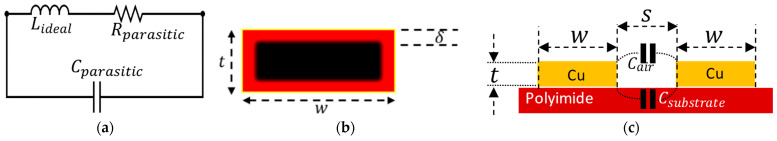
Lumped model of the elliptical planar inductor to show parasitic effects (**a**) Lumped circuit model of the planar inductor (**b**) Skin depth (δ) shown in the red area where current flows through the red area instead of the complete cross-sectional area of conductor (**c**) Parasitic capacitance $\left(C_{parasitic}\right)$ between nearby turns.

After transforming the area of the ellipse into the circular inductor, the values of the parasitic components for the elliptical planar inductor can be calculated using the existing circular inductor expressions. The lumped model of the planar inductor is shown in Figure 4a, consisting of parasitic resistance ($R_{parasitic}$), parasitic capacitance ($C_{parasitic}$), and an ideal inductor ($L_{ideal}$).

The total conductor length of the planar elliptical planar $\left(l_{ellipse}\right)$ can be calculated by Equation (11) after achieving the newer inner diameter $\left(d_{in\_c}\right)$ and outer diameter $\left(d_{out\_c}\right)$ using Equations (8) and (9).

(11) $$l_{ellipse}=\frac{\pi N\left(d_{in\_c}+d_{out\_c}\right)}{2}$$

The quality factor of the inductor plays a critical role in the inductor’s performance, and the quality factor is hugely impacted by the parasitic resistance ($R_{parasitic}).$ The value of $R_{parasitic}$ is dependent on the direct current resistance $\left(R_{DC}\right)$ and the alternating current resistance $(R_{AC}$). Equation (12) is used to compute $R_{parasitic}$.

(12) $$R_{parasitic}=R_{AC}+R_{DC}$$

Equation (13) is used to compute the DC component of the resistance $\left(R_{DC}\right)$.

(13) $$R_{DC}=\frac{\rho l_{ellipse}}{Wt}$$

where $l_{ellipse}$ is the total length of the elliptical spiral conductor, t is the trace thickness, W is the trace width, and ρ is the resistivity of the conductor.

The AC resistance ($R_{AC}$) of the planar inductor is frequency dependent and becomes significantly higher than the DC resistance $\left(R_{DC}\right)$ at higher frequencies due to the skin and proximity effects [48]. Therefore, the AC resistance ($R_{AC}$) can be computed from Equation (14).

(14) $$R_{AC}=R_{skin}+R_{proximity}$$

At higher frequencies, the alternating current flows through the outer area of the conductor rather than flowing through the complete cross-sectional area of the conductor; this effect is called the skin effect. Due to a reduction in the effective cross-sectional area, the current flow faces more resistance, which is known as the skin effect resistance $\left(R_{skin}\right)$. In Figure 4b, the red area shows the skin depth $\left(\delta \right)$ through which current flows, and the black area represents the area with no current flow. Equation (15) is used to compute the resistance due to the skin effect $\left(R_{skin}\right)$ [15].

(15) $$R_{skin}=\frac{\rho l_{ellipse}}{W\delta \left(1-e^{\frac{-t}{\delta }}\right)\left(1+\frac{t}{W}\right)}\;\mathrm{where}\;\delta =\sqrt{\frac{\rho }{\pi \mu_{o}\mu_{r}f}}$$

here $\mu_{o}\;$is the permeability constant, and its value is $4\pi \times 10^{-7}\;H/m$, $\mu_{r}$ is the relative permeability of the conductor, and f is the operational frequency.

Similar to the skin effect, the proximity effect also becomes significant at higher frequencies. At a specific frequency (crowding frequency $\left(f_{critical}\right)$), the magnetic field of the nearby turns of the planar inductor becomes significantly high and causes a nonuniform flow through the traces. This nonuniform distribution of current results in increased resistance which is known as proximity resistance $\left(R_{proximity}\right)$ and can be computed using Equation (16) [49].

(16) $$\;\;\;\;\;\;\;\;\;\;\;\;\;\;\;R_{proximity}=\frac{R_{DC}}{10}{\left(\frac{f}{f_{critical}}\right)}^{2},\;\mathrm{where}\;f_{critical}=\;\frac{3.1\left(W+S\right)\rho }{2\pi \mu_{o}W^{2}\;t}$$

The parasitic capacitance ($C_{parasitic}$) is one of the significant parasitic components and can limit the functionality of the inductor. The parasitic capacitance for the planar inductors can be computed from Equation (17) [50,51]. Total parasitic capacitance is a combined effect of capacitances between the nearby metallic traces due to the air gap between traces and underlying substrate material (polyimide).

(17) $$C_{parasitic}=C_{air}+C_{substrate}=\frac{l_{ellipse}t\epsilon_{o}}{s}(\alpha \epsilon_{air}+\beta \epsilon_{substrate})\;$$

The contributing factors of parasitic capacitances are *α* = 0.9 and *β* = 0.1. The parasitic capacitance due to the air gap $\left(C_{air}\right)$ and parasitic capacitance due to the underlying substrate ($C_{substrate}$) are shown in Figure 4c. The relative permittivities of the substrate material and air are expressed as $\epsilon_{substrate}\;$and $\epsilon_{air}$, respectively.

Once the parasitic capacitance is computed, then the self-resonance frequency$\;\left(f_{SRF}\right)$ of the planar elliptical inductor can easily be computed using the self-inductance of the elliptical inductor and parasitic capacitance. The $f_{SRF}$ is very critical while designing an inductor for specific applications with a wide range of operational frequencies. Above its self-resonance frequency, an inductor works more like a capacitor than an inductor. The $f_{SRF}\;$can be computed using Equation (18) [51].

(18) $$f_{SRF}=\frac{1}{2\pi \sqrt{L_{ellipse}C_{parasitic}}}$$

### 2.4. Fabrication

**Figure 5:**
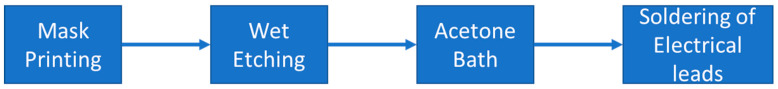
Stepwise fabrication process of elliptical planar inductors.

To validate the proposed elliptical inductor model, different elliptical planar inductors were made using a wet-etching method. In the first fabrication stage, a LaserJet printer (HPM553, HP Technology, Dublin, Ireland) was used to print the inductor mask directly onto a 50 µm thick single-sided copper-coated polyimide film (Flexible Isolating Circuit 50 Microns-Coppered 35 Microns-1 Side, CIF, Buc, France). As the next step, these copper-coated polyimide films with printed masks were attached to the plastic stand of the etching machine (PA104 Heated Bubble Etch tank, Fortex, UK). The etchant was made by combining sodium persulphate (Na₂S₂O₈) and deionized water in a 1:5 ratio. The etching was carried out inside a transparent acrylic tank with a diaphragm air pump attached to microporous tubing to produce tiny air bubbles that would help the etching process. To increase the speed of the etching process, the etchant’s temperature was set at 42 °C by using a suspended glass heater dipped inside the machine tank. The overall etching process was completed within 20–25 min. In the next step, this patterned flexible PCB was removed from the etching tank and washed with hot water. The ink particles from the patterned inductor designs were removed using an acetone bath. In the final step of the fabrication, flexible multithread copper wires were soldered on the terminal points of the inductors for electrical connections. The stepwise fabrication process is shown in Figure 5.

### 2.5. Device Validation

**Figure 6:**
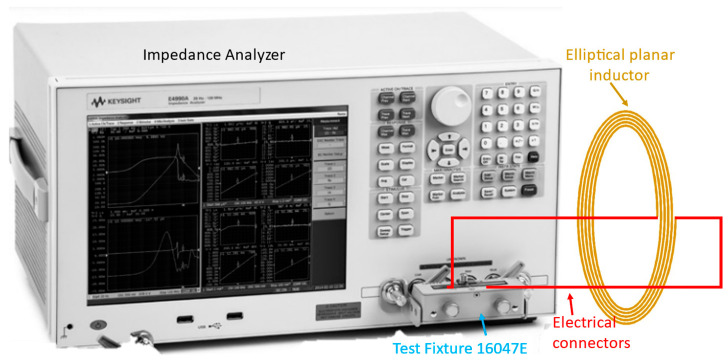
Inductance and Impedance measurement setup.

To validate the proposed model, a Keysight E4990A impedance analyzer (Keysight Technologies Inc., CA, USA) was used for the measurements of fabricated elliptical inductors. Before the measurements, the impedance analyzer was calibrated using the standard test fixture 16047E (Keysight Technologies Inc., CA, USA) (open and short calibration). The fabricated elliptical planar inductors were connected in this test fixture, as shown in Figure 6, and the inductance$\;(L_{ellipse})\;$and the real $\left(R_{ellipse}\right)$ and imaginary $(X_{ellipse})\;$parts of the impedance were measured for a range of frequencies between 20 Hz and 120 MHz (the full-scale measurement range of the E4990A). However, to validate the proposed model, the inductance measurements reported in the tables were taken at 1 MHz frequency as the equipment error is only 0.1% at 1 MHz, and it became 5–10% when the measurement frequency is >100 MHz [52].

## 3. Results

To validate and assess the estimation accuracy of the proposed elliptical model, different sets of elliptical planar inductors were fabricated. Considering the significant impact of parasitic components in varying geometries with different trace widths, trace separations, minor and major diameters, and the number of turns, a large number of inductors (*n* = 75) were fabricated. In one set, the major-to-minor ratio $\left(R\right)$ between the inner minor and inner major diameters ($d_{in\_maj}\;\mathrm{to}\;d_{in\_min})$ was kept fixed, and the trace width (*W*) and trace separation (*S*) were varied. Similarly, for another set of fabricated inductors, the ratio between the outer minor and outer major diameters ($d_{in\_maj}\;\mathrm{to}\;d_{in\_min}$) was varied while keeping the trace width and trace separation constant.

### 3.1. Estimation Results for Varying Trace Separation (S) and Trace Width (W) While Keeping the Ratio (R) between the Inner Minor Diameter to Inner Major Diameter Constant

**Figure 7:**
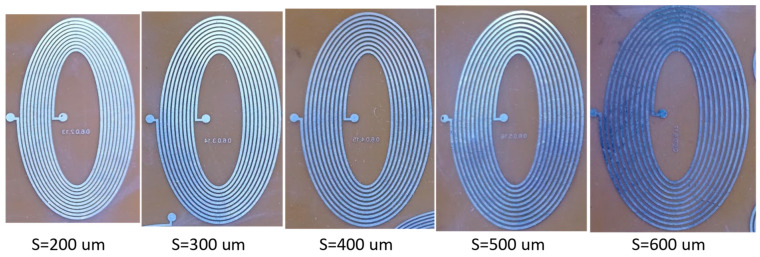
Elliptical planar inductors with a width of 600 μm and varying separation (*S*) while major-to-minor ratio (*R*) = 3 and number of turns (*N*) = 10 were kept constant.

This section details the comparison between the numerical results of the proposed model and the measured results of the fabricated elliptical planar inductors. In this comparison, the ratio between the inner minor and inner major diameters was kept fixed at 3, while the trace width and trace separation were varied between 200 µm and 600 µm for 10- and 5-turn elliptical inductors. The outer minor and outer major diameters were dependent on the combinations of trace width and trace separation. Table 2 and Table 3 show the calculated and measured results, respectively, for the 10-turn elliptical inductor. The ratio between the outer minor and outer major diameters was kept at 3. Figure 7 represents the fabricated elliptical inductors with a trace width of 600 μm and trace separation varying from 200 µm to 600 µm, while the major-to-minor ratio (*R*) and *N* are kept constant at 3 and 10, respectively.

The percentage error, as given in Equation (19), is an evaluation metric commonly used to compare estimated and experimental measurements [23,27]. The percentage error between the numerical inductance of the proposed model and measured inductance values of the elliptical planar inductor is computed using Equation (19) and given in Table 4. A maximum error value of 6.42% was found when the trace separation and trace width were 600 μm, while the minimum error was found to be 0.08% when the trace separation (*S*) and trace width (*W*) were 500 μm and 200 μm, respectively. However, the average error was 2.47%, with a standard deviation of 1.8% for the different combinations of trace separation (*S*) and width (*W*).

(19) $$\%\;Error=\left|\frac{L_{meas}-L_{cal}}{L_{meas}}\;\right|\times 100$$

**Figure 8:**
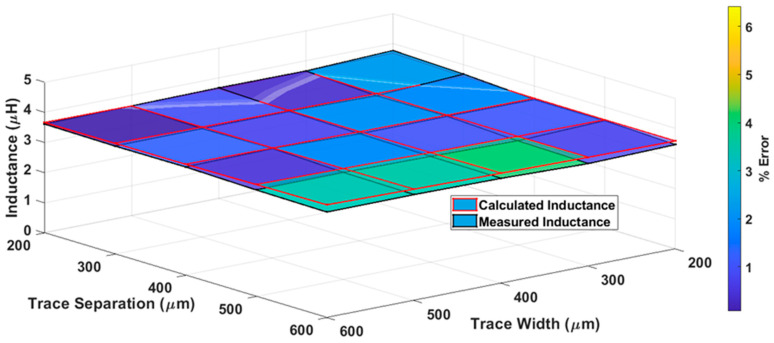
Surface plot of proposed model inductances and measured inductances of the elliptical planar inductor with a color bar to represent the percentage error for different combinations of trace separation (*S*) and trace width (*W*) while major-to-minor ratio (*R*) = 3 and number of turns (*N*) = 10.

The measured inductances (black lines) and proposed model inductances (red lines) with color bar graphs for various trace separations and trace width are represented graphically in Figure 8. It is evident from Figure 8 that the proposed model inductance and measured inductance values are approximately the same, with a percentage difference of less than 5% between the values. This difference is primarily associated with fabrication inaccuracies and measurement errors.

**Figure 9:**
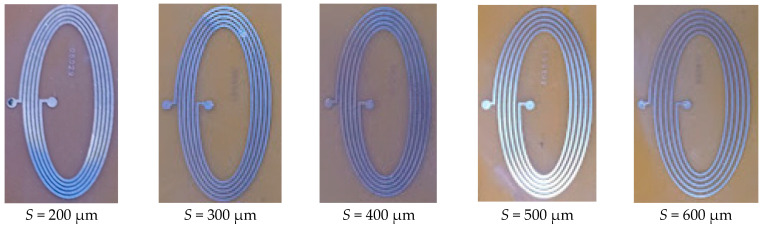
Elliptical planar inductors with trace width (*W*) of 600 μm and varying trace separation (*S*) while major-to-minor ratio (*R*) = 3 and number of turns (*N*) = 5.

To evaluate the impact of the number of turns of the elliptical inductor on varying trace separation and trace width, five-turn elliptical inductors were investigated. The fabricated elliptical inductors with five turns are shown in Figure 9. For a fair comparison with previous results, trace separation and trace width combinations were kept the same as in the last test setup. The calculated and measured inductance values are tabulated in Table 5 and Table 6, respectively. The maximum value of the calculated inductance was found for a trace width and trace separation of 200 μm, and the minimum inductance value was seen for a trace width of 600 μm and trace separation of 400 μm.

The measured inductance of elliptical planar inductors is tabulated in Table 5. It can be observed from Table 6 that the measured inductance values are lower than the calculated inductance values. The measured inductance was higher due to the additional copper leads soldered with the fabricated inductor for electrical connections with an impedance analyzer. The maximum measured inductance is 1 μH, while the minimum measured inductance is observed to be 0.833 μH for a trace separation of 600 μm and a trace width of 500 μm.

The percentage error between the numerical inductance of the proposed model and the measured inductance values of the elliptical planar inductor is given in Table 7. It can be observed from Table 7 that the maximum error was observed to be 9.93% for a trace separation of 300 μm and a trace width of 500 μm. The minimum error was observed to be 0.59% for a trace separation and trace width of 200 μm. Moreover, the average error was 4.85%, with a standard deviation of 3.18% for the different combinations of trace separation and trace width for a fixed number of turns (5) and fixed major-to-minor ratio (3). Further, it can be observed from Table 7 that the average percentage error is slightly higher for 5-turn elliptical inductors compared to 10-turn elliptical inductors. This error is higher as fewer-turn inductors are more prone to variability in fabrication and measurement phases than large-turn inductors.

**Figure 10:**
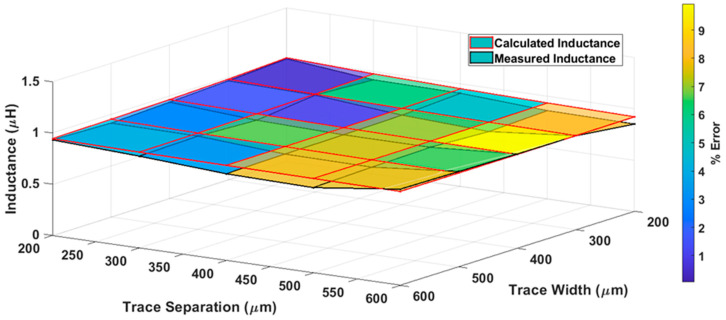
Surface plot of the numerical inductance of the proposed model and measured inductance of the elliptical planar inductor with a color bar to represent the percentage error for different combinations of trace separation (*S*) and trace width (*W*) while major-to-minor ratio (*R*) = 3 and number of turns (*N*) = 5.

The measured inductances (black lines) and numerical inductances of the proposed model (red lines) with color bar graphs for various trace separations and trace width have been represented graphically in Figure 10. It is evident from Figure 10 that the numerical inductance of the proposed model and measured inductance values are approximately the same, with a percentage difference of less than 8% between the values. 

### 3.2. Estimation Results for Varying Ratios of Inner Minor Diameter to the Inner Major Diameter between 1 to 5 While Keeping Trace Separation and Width Fixed at 200 μm

**Figure 11:**
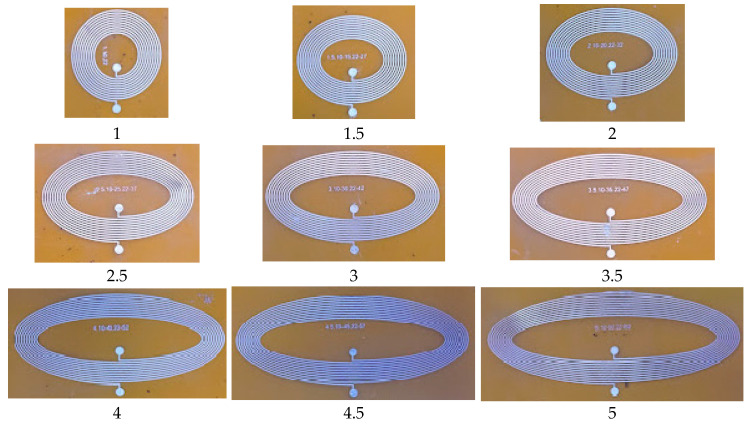
Fabricated elliptical planar inductors with varying major-to-minor ratio (*R*) values of between one and five with an interval of 0.5, while trace separation (*S*) = trace width (*W*) = 200 μm.

To investigate the effect of the varying major-to-minor ratio between one and five, elliptical inductors of different sizes were fabricated and tested, as shown in Figure 11. In all planar inductors, the number of turns, trace width, and trace separation were kept fixed at 10, 200 μm, and 200 μm, respectively. The inner minor diameter was set to 5 mm, while the inner major diameter was varied from 5 to 25 mm to achieve major-to-minor ratio (*R*) values of between one and five. The numerical inductance $\left(L_{cal}\right)$ is calculated using the proposed model mentioned in Section 2.1, whereas the measured inductance ($L_{meas}$) is measured using the impedance analyzer. The numerical inductance of the proposed model and measured inductance values are listed in Table 8. To calculate the difference between the numerical inductance of the proposed model and measured inductance values, the absolute percentage error has been calculated and is tabulated in Table 8.

It can be observed from Table 8 that the maximum error was observed to be 6.38% for the major-to-minor ratio of 5, and the minimum error was observed to be 0.45% for the major-to-minor ratio of 2.5. Moreover, the average error between the calculated and measured inductances was observed to be 3.61%, with a standard deviation of 2.11%. The major contributors to this error are the fabrication, measurement, and methodology to estimate the inductance of the planar elliptical inductors.

**Figure 12:**
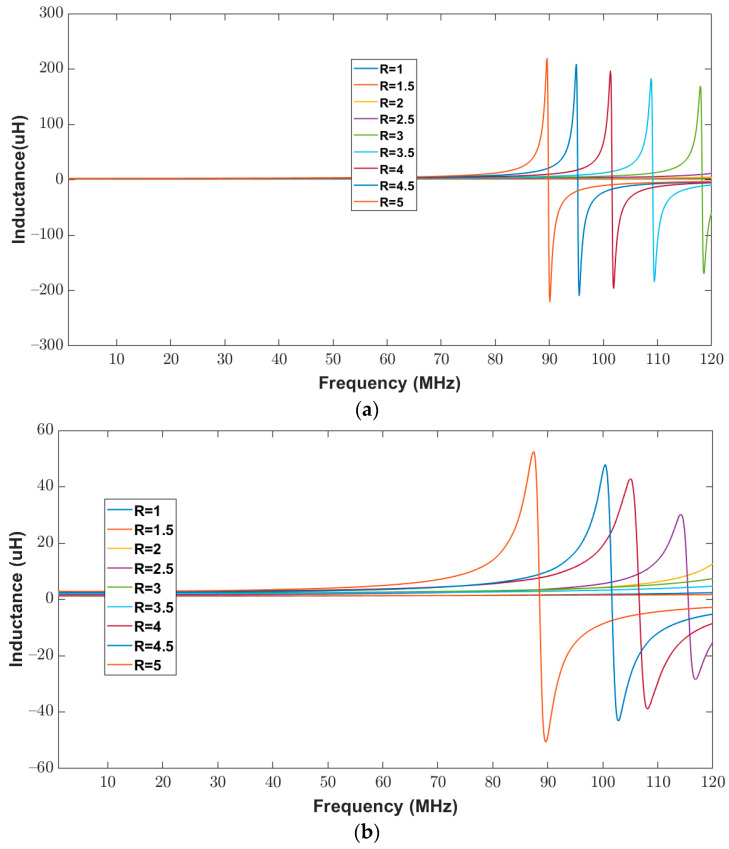
Inductance of elliptical planar inductors for the full range of frequencies from 20 Hz to 120 MHz with varying major-to-minor ratio values while trace separation (*S*) and trace width (*W*) were kept constant at 200 μm (**a**) Computed response using proposed model (**b**) Measured response from the impedance analyzer.

To validate the proposed model, the inductance of the elliptical inductor was calculated for a frequency range of 20 Hz to 120 MHz. The measured inductance of all elliptical inductors was recorded using an impedance Analyzer. Figure 12a,b show the numerical inductance of the proposed model and the measured inductance of the elliptical inductors, respectively. It can be observed from Figure 12 that, for all combinations of the major-to-minor ratio (1 to 5), the trends in the measured and the calculated inductance values look similar. These similar trends between the calculated and measured values validate the proposed model. Moreover, the percentage difference between the calculated and measured inductance values has been calculated for the observed frequency range, and the values are tabulated in Table 8.

The self-resonance frequency ($\;f_{SRF})$ is an important performance metric to analyze the behavior of an inductor as the parasitic capacitance dominates at frequencies higher than the self-resonance frequency. Thus, while designing an inductor for higher frequencies, it is not enough to choose the correct inductance but also essential to use an inductor with a self-resonance frequency substantially lower than the $f_{SRF}$. Therefore, to analyze the proposed numerical model and measured elliptical planar inductors, this study has compared $f_{SRF}$ values calculated using our model with experimental data. It can be noted from Table 9 that the maximum deviation between calculated ($f_{SRF\_cal}$) and measured self-resonance frequency ($f_{SRF\_meas}$) values is observed to be 6.72% for a trace separation and width of 200 μm and a major-to-minor ratio of 4.5. The N/A in this Table 9 represents that the percentage error is not available as the self-resonance frequency was higher than the frequency range (>120 MHz) of the impedance analyzer.

**Figure 13:**
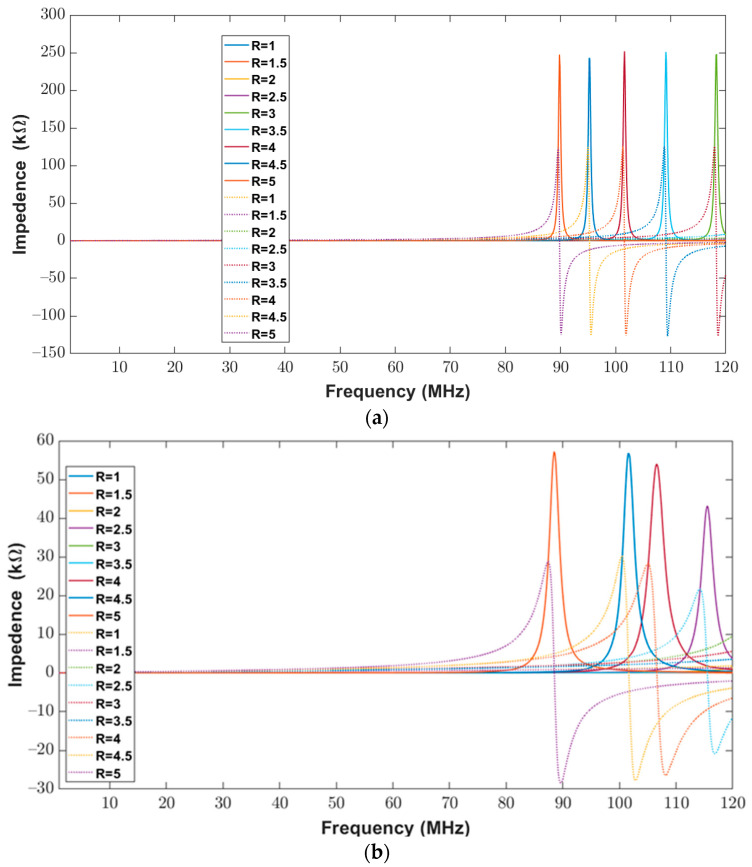
Impedance (Real (solid lines) and Imaginary (dotted lines) components) of elliptical planar inductors for the full range of frequencies from 20 Hz to 120 MHz with varying major-to-minor ratios while trace separation (*S*) and trace width (*W*) were kept constant to 200 μm (**a**) Computed response using proposed model (**b**) Measured response from the impedance analyzer.

The impedance (real and imaginary) of the elliptical planar inductor calculated from the proposed model was compared with measured values from the impedance analyzer over the frequency range of 20 Hz to 120 MHz. The calculated and measured impedance results are shown in Figure 12b and Figure 13a. In both Figure 13a,b, the real part of the impedance is shown with solid lines, whereas the imaginary part is shown with dotted lines. It can be observed from Figure 12b and Figure 13a that the real and imaginary impendence profiles for both the calculated and measured results of the elliptical inductors are similar.

### 3.3. Estimation Results for Varying Ratios of Inner Minor Diameter to the Inner Major Diameter between 1 to 5 While Keeping Trace Separation and Width Fixed at 300 μm

To validate the impact of varying trace width and separation for varying major-to-minor ratios of one to five, the trace width and separation were set to 300 μm while the number of turns was kept fixed at 10. The inner minor diameter was set to 10 mm, while the inner major diameter was varied from 10 to 50 mm to achieve major-to-minor ratio values of between one and five. The calculated and measured inductance values are listed in Table 10. To compute the difference between the calculated and measured inductance values, the absolute percentage error has been calculated and is tabulated in Table 10.

The maximum error was observed to be 7.85% for a major-to-minor ratio of five, whereas the minimum error was observed to be 0.28% for a major-to-minor ratio of three. The average error between the calculated and measured values was observed to be 4.19%, with a standard deviation of 2.39%. As explained previously, the errors arise mainly from the fabrication, measurement, and methodology of estimating the inductance of the planar elliptical inductors.

**Figure 14:**
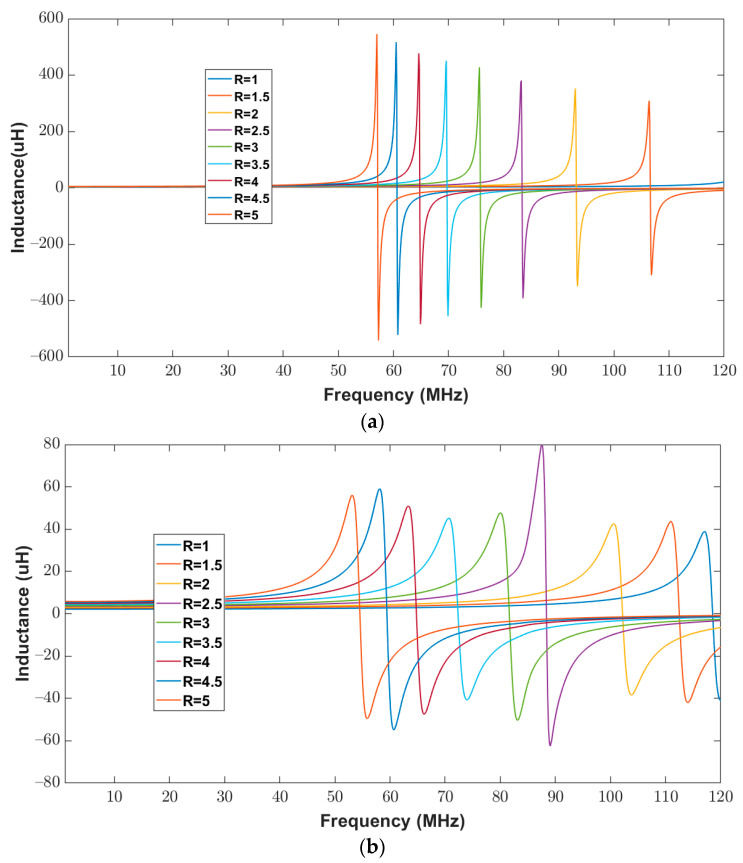
Inductance of elliptical planar inductors for the full range of frequencies from 20 Hz to 120 MHz with varying major-to-minor ratios while trace separation (*S*) and trace width (*W*) were kept constant at 300 μm (**a**) Computed response using proposed model (**b**) Measured response from the impedance analyzer.

To validate the proposed model, the inductance of the elliptical inductor was computed for a frequency range of 20 Hz to 120 MHz. The measured inductance of all elliptical inductors was recorded using an impedance analyzer. Figure 14a,b show the calculated and measured inductance of the elliptical inductors, respectively. It can be observed from Figure 14, that for all combinations of major-to-minor ratios (one to five), the trends in the measured and the calculated inductance values look similar. One apparent outlier in Figure 14b may have been affected by interference during measurement. However, the trend remains consistent with other data points.

It is evident from Table 11 that the maximum deviation between the calculated ($f_{SRF\_cal}$) and measured self-resonance frequency ($f_{SRF\_meas}$) values is 9.55% for the trace separation and width of 300 μm and a major-to-minor ratio of two, while the average error was 4.88% with a standard deviation of 3%.

**Figure 15:**
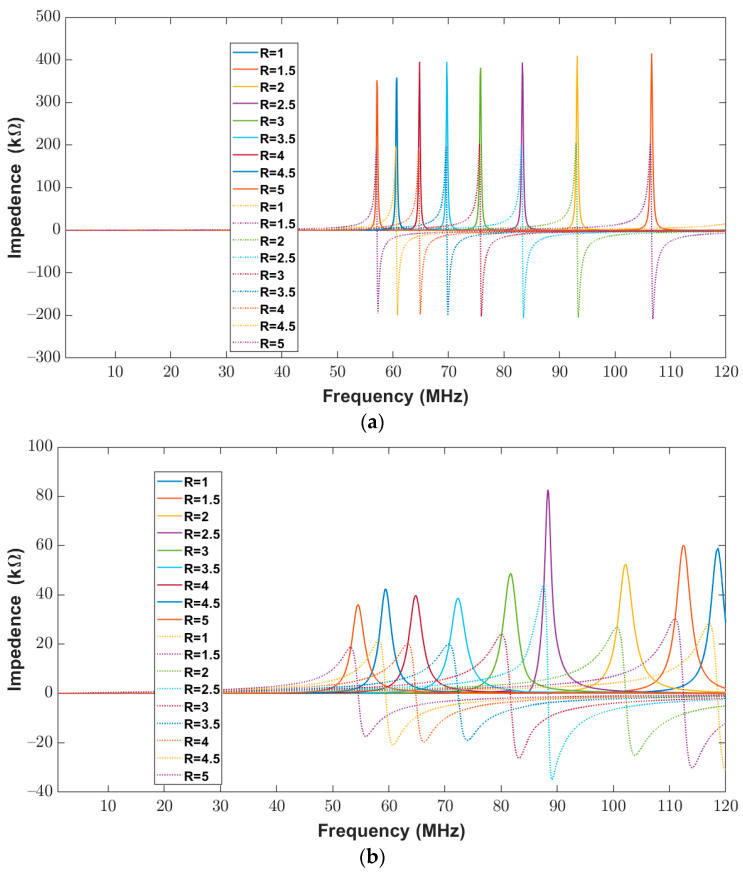
Impedance (Real (solid lines) and Imaginary (dotted lines) components) of elliptical planar inductors for the full range of frequencies from 20 Hz to 120 MHz with varying major-to-minor ratios while trace separation (*S*) and trace width (*W*) were kept constant at 300 μm (**a**) Computed response using proposed model (**b**) Measured response from the impedance analyzer.

To further validate the proposed model, a comparison between the numerical impedance of the proposed model and the measured impedance for the different major-to-minor ratios is made. During this analysis, the trace width and trace separation were kept constant at 300 μm. The calculated and measured impedance results for a frequency range of 20 Hz to 120 MHz are shown below in Figure 15a,b. The real part of the impedance is shown with solid lines, whereas dotted lines represent the imaginary part of the impedance. The results show that the profiles of real and imaginary components are similar for both the calculated and the measured results.

### 3.4. Estimation Results for Varying Ratios of Inner Minor Diameter to the Inner Major Diameter between 1 to 5 While Keeping Trace Separation and Width Fixed at 400 μm

To further analyze the impact of varying trace width and separation for varying major-to-minor ratios of one to five, the trace width and separation were set to 400 μm while the number of turns was kept at 12. The inner minor diameter was set to 12 mm, while the inner major diameter was varied from 12 to 60 mm to achieve major-to-minor ratio values of between one and five. To compute the difference between the calculated and measured inductance values, the absolute percentage error has been calculated and is tabulated in Table 12.

Table 12 shows that the maximum error was 5.31% for a major-to-minor ratio of five, whereas the minimum error was 0.03% for a major-to-minor ratio of three. The average error between the calculated and measured values was observed to be 2.22%, with a standard deviation of 1.82%. As stated earlier, the errors arise mainly from the fabrication, measurement, and methodology of estimating the inductance of planar elliptical inductors.

**Figure 16:**
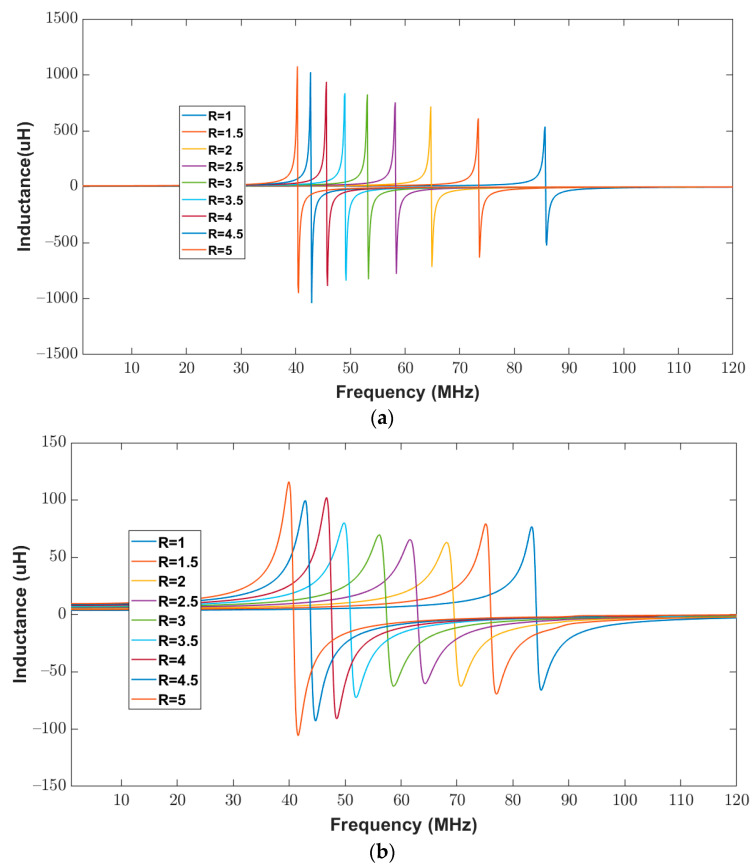
Inductance of elliptical planar inductors for the full range of frequencies from 20 Hz to 120 MHz with varying major-to-minor ratios while trace separation (*S*) and trace width (*W*) were kept constant at 400 μm (**a**) Computed response using proposed model (**b**) Measured response from the impedance analyzer.

For further validation, the inductance of the elliptical inductor was computed and measured for a frequency range of 20 Hz to 120 MHz. Figure 16a,b show the calculated and measured inductance of elliptical inductors, respectively. It can be observed from Figure 16 that for all combinations of major-to-minor ratio values (one to five), the trends in the measured and the calculated inductance results look similar.

As previously discussed, the self-resonance frequency is a critical parameter when designing an inductor. Thus, the measured and calculated self-resonance were compared and listed in Table 13, and a maximum deviation of 7.89% was noticed for a major-to-minor ratio of 2.5. However, the average error was 4.17%, with a standard deviation of 2.67%.

**Figure 17:**
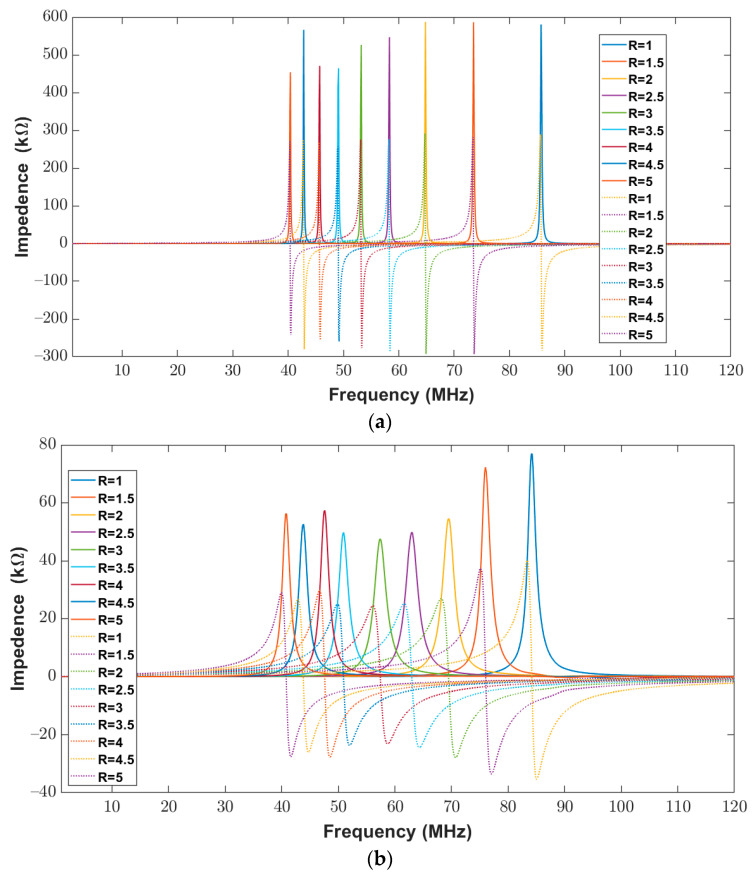
Impedance (Real (solid lines) and Imaginary (dotted lines) components) of elliptical planar inductors for the full range of frequencies from 20 Hz to 120 MHz with varying major-to-minor ratios while trace separation (*S*) and trace width (*W*) were kept constant at 400 μm (**a**) Computed response using proposed model (**b**) Measured response from the impedance analyzer.

The real and imaginary impedance components are shown in Figure 17a,b when the trace width and trace separation were kept constant at 400 μm for varying major-to-minor ratio values of between one and five. It is evident from Figure 17a,b that there was a similar response for the calculated and measured impedances of elliptical inductors.

## 4. Discussion

In this study, a numerical model of a planar elliptical inductor has been presented that uses an area transformation technique to estimate inductor parameters from a circular model. During the transformation, the minor and major inner and minor and major outer diameters of the elliptical planar inductor were transformed into the inner diameter and outer diameter of the circular planar inductor. After the transformation, the new inner diameter and outer diameter were used for further calculations of the inductance, impedance, self-resonance frequency, and other parameters of the elliptical planar inductor. To validate the proposed model, several elliptical planar inductors were fabricated. The measured and proposed numerical model results were compared to assess the accuracy of the proposed model for planar elliptical inductors. 

**Figure 18:**
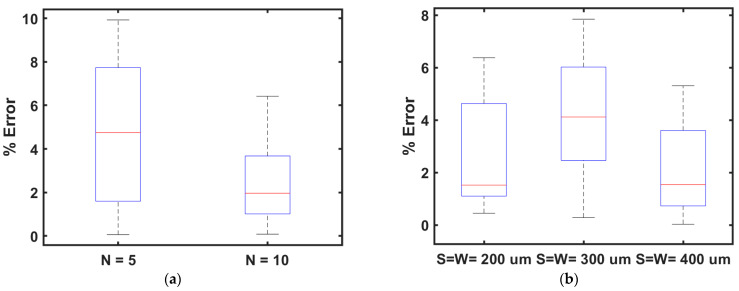
(**a**) Boxplot of the percentage error when trace width and separation were varied between 200 μm and 600 μm for fixed major-to-minor inner diameter ratio (*R*) = 3 for number of turns *N* = 5 and *N* = 10 (**b**).

The estimated inductor parameters using the proposed model showed an excellent match with the measured values from a large batch of fabricated inductors. The proposed model was validated for robustness using different combinations of trace widths, trace separation, and other geometrical features. The trace width and trace separation were varied between 200 μm to 600 μm. In the first step, the trace separation and trace width were varied for 10- and 5-turn inductors while the major-to-minor axis ratio was kept constant at three. Figure 18 represents the boxplot of percentage error between the measured and calculated inductances using the proposed model for the elliptical inductor when trace width and separation were varied between 200 μm and 600 μm. The ratio between the major and minor inner diameters was kept fixed at three while the number of turns was set to 5 and 10. From Figure 18a, it is clear that the median and variation of the percentage error were higher for *N* = 5 compared to the percentage error for *N* = 10. Table 14 shows the average percentage error and standard deviation of all measurement scenarios. From Table 14, it is evident that in all cases, the average percentage error was less than 5%, and the maximum measured standard deviation was 3.18%. 

In this investigation, it was observed that the average error of inductance for the measured and proposed numerical model was approximately two times higher for inductors with fewer turns (*N* = 5) than that for 10 turns. The standard deviation was found to be approximately two times higher for 5-turn inductors than for 10-turn inductors. This higher average error and standard deviation of percentage error in smaller inductors were because smaller inductors are more prone to show variation during the fabrication, measurement, and designing process. A very small error due to calibration or measurement will cause a higher percentage error for small inductors than for large inductors.

In the next set of experiments, the major-to-minor ratio was varied between one and five while the trace separation and trace width were kept at 200 μm, 300 μm, and 400 μm. Figure 18b represents the boxplots of percentage errors for this set of inductors. Table 14 shows percentage errors of 3.61%, 4.19%, and 2.22% when the trace width and separation were 200 μm, 300 μm, and 400 μm, respectively. Moreover, the standard deviation was found to be 2.11%, 2.39%, and 1.82% when trace width and separation were 200 μm, 300 μm, and 400 μm, respectively. This investigation observed that the average error and standard deviation were smaller when the trace width and separation were kept at 400 μm. From all sets of experiments, it can be seen clearly that the maximum error was observed to be 4.85%, and the minimum average error was observed to be 2.22%, while the overall average error was 3.47%. The maximum and minimum standard deviations were 3.18% and 1.80%, respectively, while the average standard error was 2.26%.

For comparison with existing methods, a summary of the state-of-the-art approaches is presented in Table 15. The limitations associated with each approach are also listed in Table 15. It can be observed that some of the expressions are very complex and demand high computational power and resources to evaluate the inductance of planar inductors. Most of the approaches listed in Table 15 are only suitable for symmetrical planar inductor computation. Moreover, the accuracy is also dependent on the design parameters such as trace width, trace separation, number of turns, and inner and outer diameters. The fourth column in Table 15 shows the absolute percentage error between the inductance values computed using the expression and finite element simulator. These errors tend to increase when compared with the experimental data, as experimental results may also vary due to variations in the fabrication process and measurement setup. The percentage error reported in this study between the proposed numerical model and experimental results is under 5%. This is a collective error due to the fabrication, measurement, and estimation error of the model of the planar elliptical inductor. Using a similar area transformation technique, the inductance and other parasitic components can be computed for other semi-symmetrical shapes without performing high-intensity computational power and complex mathematical modeling. The proposed model is computationally simple as shown in Equations (8)–(10). In terms of computational complexity, it takes only 5.7 ms on average to compute the inductance of a single planar inductor using the proposed model and MATLAB 2020b running on a desktop computer (Processor (Intel Core i5 CPU at 1.60 GHz 2.11 GHz), RAM 8 GB, etc.).

Overall, a small error and small standard deviation between the experimental and numerical results have been observed; however, the main limitation of this work was the variation in the fabrication process, especially when there are fine traces in the design of the elliptical planar inductor. As mentioned earlier, smaller inductors are more prone to error as the parasitic effect due to measurement setup can also change the actual values. This variation can cause an overall increase in the error between the experimental and numerical results. The proposed model in this study is based on the current sheet expression for the planar inductor model. The current sheet expression shows a 2–3% error when the trace width and separation of the planar inductor are relatively similar. This error becomes 8% when the trace separation is less than or equal to three times the trace width, which could also limit the accuracy of the proposed model. Another limitation is the impedance analyzer frequency range of 20 Hz to 120 MHz, as a smaller inductor shows the response in the higher frequencies >120 MHz; also, the measurement error increases when the measurement frequency is >100 MHz.

## 5. Conclusions

In this study, a numerical model is developed to calculate the inductance of the elliptical planar inductor and its parasitic components. An area transformation technique from an elliptical to a circular shape was used to adapt the circular planar inductor formulas. The proposed numerical model was validated for various combinations of the number of turns, trace width, trace separation, and different inner and outer diameters of the elliptical planar inductor. For the validation, a large batch of elliptical planar inductors (*n* = 75) were designed, fabricated, and measured to assess the estimation accuracy and robustness of the model. The overall average error between the measured and proposed numerical model results was less than 5%, with a standard deviation of less than 3.18%. The main factors for a higher variation in the measured results were the limitations in the fabrication process, as masks were directly printed on the flexible copper-coated sheets using a LaserJet printer, which has a lower resolution on this type of print media. Nevertheless, an excellent match of inductor parameters between the model estimates and the measured values suggests that the proposed model is a good candidate for modeling and designing elliptical planar inductors.

In future studies, the planar inductors can be fabricated using laser technology to achieve less variation and high accuracy. Using this approach, elliptical planar inductors can be designed, optimized, and fabricated for several applications, including implantable devices. In the future, these elliptical planar inductors can be integrated with passive sensing capacitive elements to realize LC wireless sensors. The elliptical inductors can be folded into a compact shape, making them suitable for a catheter delivery system to remote and narrow implantation sites. The proposed approach of the area transformation technique can be used to compute the inductance and other parasitic components for other semi-symmetrical shapes without performing high-intensity computational power and complex mathematical modeling.

## Figures and Tables

**Table 1:** Geometrical coefficients for current sheet expression [23].

Layout	$C_{1}$	$C_{2}$	$C_{3}$	$C_{4}$
Circular	1	2.46	0	0.2
Octagonal	1.07	2.29	0	0.19
Hexagonal	1.09	2.23	0	0.17
Square	1.27	2.07	0.18	0.13

**Table 2:** Inductance of elliptical inductors calculated from the proposed model for different combinations of trace separation (*S*) and trace width (*W*) while major-to-minor ratio (*R*) = 3 and number of turns (*N*) = 10.

Calculated Inductance (μH) of Elliptical Inductor Models (Number of Turns (*N*) = 10, Major-to-Minor Ratio (*R*) = 3)						
Test Parameters		Trace Separation (µm)				
		200	300	400	500	600
**Trace Width** **(µm)**	**200**	3.69	3.65	3.63	3.64	3.66
	**300**	3.62	3.61	3.62	3.64	3.67
	**400**	3.59	3.60	3.62	3.65	3.68
	**500**	3.58	3.60	3.63	3.67	3.71
	**600**	3.58	3.61	3.65	3.69	3.74

**Table 3:** Measured inductances of elliptical planar inductors for different combinations of trace separation (*S*) and trace width (*W*) while major-to-minor ratio (*R*) = 3 and number of turns (*N*) = 10.

Measured Inductance (μH) of Fabricated Elliptical Inductors (Number of Turns (*N*) = 10 Major-to-Minor Ratio (*R*) = 3)						
Test Parameters		Trace Separation (µm)				
		200	300	400	500	600
**Trace Width** **(µm)**	**200**	3.77	3.66	3.67	3.64	3.61
	**300**	3.69	3.54	3.59	3.59	3.58
	**400**	3.55	3.56	3.55	3.63	3.59
	**500**	3.55	3.45	3.50	3.54	3.53
	**600**	3.46	3.40	3.48	3.51	3.50

**Table 4:** The percentage error between the numerically calculated and measured inductances of elliptical planar inductors after using the proposed model for different combinations of trace separation (*S*) and trace width (*W*) while major-to-minor ratio (*R*) = 3 and number of turns (*N*) = 10.

% Error in Calculated and Measured Inductances (Number of Turns (*N*) = 10, Major-to-Minor Ratio (*R*) = 3)						
Test Parameters		Trace Separation (µm)				
		200	300	400	500	600
**Trace Width** **(µm)**	**200**	2.17	0.39	0.95	0.08	1.26
	**300**	1.82	1.96	0.75	1.28	2.46
	**400**	1.09	1.03	1.86	0.47	2.62
	**500**	0.75	4.11	3.53	3.44	4.84
	**600**	3.31	5.80	4.59	4.92	6.42

**Table 5:** Inductance of elliptical inductors calculated from the proposed model for different combinations of trace separation (*S*) and trace width (*W*) while major-to-minor ratio (*R*) = 3 and number of turns (*N*) = 5.

Calculated Inductance (μH) of Elliptical Inductor Models (Number of Turns (*N*) = 5, Major-to-Minor Ratio (*R*) = 3)						
Test Parameters		Trace Separation (µm)				
		200	300	400	500	600
**Trace Width** **(µm)**	**200**	1.01	0.982	0.964	0.953	0.945
	**300**	0.975	0.958	0.947	0.940	0.935
	**400**	0.952	0.941	0.934	0.930	0.928
	**500**	0.935	0.928	0.924	0.922	0.922
	**600**	0.922	0.919	0.917	0.917	0.918

**Table 6:** Measured inductances of elliptical planar inductors for different combinations of trace separation (*S*) and trace width (*W*) while major-to-minor ratio (*R*) = 3 and number of turns (*N*) = 5.

Measured Inductance (μH) of Fabricated Elliptical Inductors (Number of Turns (*N*) = 5, Major-to-Minor Ratio (*R*) = 3)						
Test Parameters		Trace Separation (µm)				
		200	300	400	500	600
**Trace Width** **µm)**	**200**	1.00	0.966	0.938	0.915	0.933
	**300**	0.916	0.948	0.884	0.910	0.896
	**400**	0.907	0.875	0.864	0.857	0.846
	**500**	0.860	0.836	0.864	0.851	0.833
	**600**	0.851	0.918	0.921	0.931	0.940

**Table 7:** The percentage error between the numerically calculated and measured inductances of elliptical planar inductors after using the proposed model for different combinations of trace separation (*S*) and trace width (*W*) while major-to-minor ratio (*R*) = 3 and number of turns (*N*) = 5.

% Error in Calculated and Measured Values (Number of Turns (*N*) = 5, Major-to-minor ratio (*R*) = 3)						
Test Parameters		Trace Separation (µm)				
		200	300	400	500	600
**Trace Width** **(µm)**	**200**	0.59	1.61	2.73	3.96	1.30
	**300**	6.10	1.08	6.64	3.15	4.19
	**400**	4.75	7.02	7.48	7.81	8.81
	**500**	8.04	9.93	6.50	7.73	9.67
	**600**	7.75	0.06	0.45	1.54	2.36

**Table 8:** Key results of elliptical planar inductors for varying ratios when trace separation (*S*) and trace width (*W*) were kept constant at 200 μm.

$\;d_{in\_min}\;(\mathrm{mm})$	$\;d_{in\_maj}\;(\mathrm{mm})$	$\;d_{out\_min}\;(\mathrm{mm})$	$\;d_{out\_maj}\;(\mathrm{mm})$	*R*	$L_{cal}\;(\mu H)$	$L_{meas}\;(\mu H)$	% Error
5	5	13	13	1	1.02	1.00	2.31
5	7.5	13	15.5	1.5	1.23	1.22	0.74
5	10	13	18	2	1.43	1.41	1.22
5	12.5	13	20.5	2.5	1.62	1.63	0.45
5	15	13	23	3	1.81	1.84	1.52
5	17.5	13	25.5	3.5	1.99	2.02	1.47
5	20	13	28	4	2.16	2.26	4.58
5	22.5	13	30.5	4.5	2.33	2.45	4.79
5	25	13	33	5	2.50	2.66	6.38

**Table 9:** Self-resonance frequency of elliptical planar inductors for varying major-to-minor ratios while trace separation (*S*) and trace width (*W*) were kept constant at 200 μm.

*R*	1	1.5	2	2.5	3	3.5	4	4.5	5
$f_{SRF\_cal}$ (MHz)	>120	>120	>120	>120	118.2	109.1	101.6	95.2	89.8
$f_{SRF\_meas}$ (MHz)	>120	>120	>120	>120	>120	115.5	106.4	101.6	88.4
% Error	N/A	N/A	N/A	N/A	N/A	5.87	4.72	6.72	1.56

**Table 10:** Key results of elliptical planar inductors for varying major-to-minor ratios when trace separation (*S*) and trace width (*W*) were kept constant at 300 μm.

$\;d_{in\_min}$ (mm)	$\;d_{in\_maj}$ (mm)	$\;d_{out\_min}$ (mm)	$\;d_{out\_maj}$ (mm)	*R*	$L_{cal}$ (μH)	$L_{meas}$ **(μH)**	% Error
10	10	22	22	1	1.97	1.85	6.24
10	15	22	27	1.5	2.41	2.29	5.19
10	20	22	32	2	2.83	2.72	3.72
10	25	22	37	2.5	3.23	3.14	2.73
10	30	22	42	3	3.61	3.62	0.28
10	35	22	47	3.5	3.98	4.05	1.66
10	40	22	52	4	4.34	4.52	4.12
10	45	22	57	4.5	4.68	4.96	5.95
10	50	22	62	5	5.02	5.41	7.85

**Table 11:** Self-resonance frequency of elliptical planar inductors for varying major-to-minor ratios when trace separation (*S*) and trace width (*W*) were kept constant at 300 μm.

*R*	1	1.5	2	2.5	3	3.5	4	4.5	5
$f_{SRF\_cal}$ (MHz)	>120	106.6	93.2	83.3	75.7	69.7	64.8	60.6	57.1
$f_{SRF\_meas}$ (MHz)	118.3	112.3	102.1	88.3	81.5	72.2	64.7	59.3	54.5
% Error	N/A	5.35	9.55	6.00	7.66	3.59	0.15	2.15	4.55

**Table 12:** Key results of elliptical planar inductors for varying major-to-minor ratios when trace separation (*S*) and trace width (*W*) were kept constant at 400 μm.

$\;d_{in\_min}\;(\mathrm{mm})$	$\;d_{in\_maj}\;(\mathrm{mm})$	$\;d_{out\_min}\;(\mathrm{mm})$	$\;d_{out\_maj}\;(\mathrm{mm})$	*R*	$L_{cal}\;(\mu H)$	$L_{meas}\;(\mu H)$	% Error
12	12	31.2	31.2	1	3.52	3.40	3.36
12	18	31.2	37.2	1.5	4.23	4.18	1.40
12	24	31.2	43.2	2	4.92	4.88	0.82
12	30	31.2	49.2	2.5	5.59	5.56	0.45
12	36	31.2	55.2	3	6.23	6.23	0.03
12	42	31.2	61.2	3.5	6.85	6.96	1.54
12	48	31.2	67.2	4	7.45	7.66	2.68
12	54	31.2	73.2	4.5	8.04	8.39	4.34
12	60	31.2	79.2	5	8.62	9.08	5.31

**Table 13:** Self-resonance frequency of elliptical planar inductors for varying major-to-minor ratios when trace separation (*S*) and trace width (*W*) were kept constant at 400 μm.

*R*	1	1.5	2	2.5	3	3.5	4	4.5	5
$f_{SRF\_cal}$ (MHz)	85.7	73.5	64.8	58.3	53.2	49.1	45.7	42.8	40.4
$f_{SRF\_meas}$ (MHz)	84.1	75.9	69.3	62.9	57.3	50.7	47.4	43.7	40.7
% Error	1.87	3.27	6.94	7.89	7.71	3.26	3.72	2.10	0.74

**Table 14:** Average percentage error and standard deviation of percentage error of inductance between the proposed model and measured inductances.

% Error	Average % Error and Standard Deviation of % Error for Inductance					
	*W* and *S* Varied from 200 μm to 600 μm and *R* = 3		*R* Varied between 1 to 5 for *N* = 10			Overall
	*N* = 5	*N* = 10	*S = W* = 200 μm	*S = W* = 300 μm	*S = W* = 400 μm	
Average	4.85	2.47	3.61	4.19	2.22	3.47
Standard Deviation	3.18	1.80	2.11	2.39	1.82	2.26

**Table 15:** Comparison of state-of-the-art approaches for computing the inductance of planar inductors.

Technique	Methodology/Expression/Simulator	Limitations	% Error
$\mathrm{Grover}\;\mathrm{Expression}\;(L_{gmd2})$ [37]	$L_{gmd2}=L_{self}+M_{+}-M_{-}$ $L_{self}=0.002l\left[\ln\left(\frac{2l}{W+t}\right)+0.50049+\frac{W+t}{3l}\right]$ $L_{self}$ is the self-inductance of the single current-carrying electrode, *W* and *t* are the trace thickness and width, and *l* is the length of the conductor, $\;\left|M_{+}\right|=\left|M_{-}\right|=2lQ\;Q=\ln\left[\frac{l}{gmd}+\sqrt{1+{\left(\frac{l}{gmd}\right)}^{2}}\right]+\frac{gmd}{l}-\sqrt{1-{\left(\frac{gmd}{l}\right)}^{2}}$ Where *gmd* is the geometric mean distance between two conductors and can be computed using the below equation. *P* is the pitch of the coil. $\;gmd=ln\left(P\right)-\left(\frac{1}{12{\left(\frac{P}{w}\right)}^{2}}+\frac{1}{60{\left(\frac{P}{w}\right)}^{4}}+\frac{1}{168{\left(\frac{P}{w}\right)}^{6}}+\frac{1}{360{\left(\frac{P}{w}\right)}^{8}}+\frac{1}{660{\left(\frac{P}{w}\right)}^{10}}+\ldots \right)$	Only suitable when the number of turns is an integer Not suitable for quarter turn Only suitable for symmetrical inductors Perform complex computation It takes a long time to evaluate the inductance.	12.9
Wheeler Expression $\left(L_{wh}\right)$ [40]	$L_{wh}=\frac{N^{2}r^{2}}{8r+11\Delta }$ Here *r*$\;\mathrm{is}\;\mathrm{the}\;\mathrm{radius}\;\mathrm{of}\;\mathrm{the}\;\mathrm{coil},\Delta =\frac{\left(d_{out\;}-d_{in}\right)}{2}$	Only accurate for circular solenoid coils Error increases with the increase in trace width	5–20
Modified Wheeler Expression $\left(L_{m\_wh}\right)$ [23]	$L_{m\_wh}=k_{1}\mu_{o}\frac{N^{2}d_{avg}}{1+\tau k_{2}}$ Where$\;d_{avg}=\frac{\left(d_{in}+d_{out\;}\right)}{2}$$,\;\tau =\frac{\left(d_{out\;}–\;d_{in}\right)}{\left(d_{out\;}+d_{in}\right)}$$\;\mathrm{and}\;k_{1}$$\;\mathrm{and}\;k_{1}$ are geometry dependent	Only suitable for symmetrical inductors	9.8
Current Sheet Expression $\left(L_{sheet}\right)$ [23]	$L_{sheet}=\frac{µN^{2}d_{avg}C_{1}}{2}\left(\ln\left(C_{2}/\tau \right)+C_{3}\tau +C_{4}\tau^{2}\right)$ Where$\;C_{i\;}$ is geometry dependent	If S≈W then the error is 2–3%S<=3 W then the error is 8%	9.9
Monomial Expression $\left(L_{mon}\right)$ [23]	$L_{mon}=\beta d_{out}^{\alpha_{1}}W^{\alpha_{2}}d_{avg}^{\alpha_{3}}N^{\alpha_{4}}S^{\alpha_{5}}$ Here$\;\alpha_{i}$ and *β* are geometry dependent	Only suitable for symmetrical inductors	9
Crols Expression $\left(L_{Crols}\right)$ [38]	$L_{Crols}=K_{L}\frac{A_{r}{}^{\frac{3}{2}}}{W^{2}}\;\eta_{A_{r}}^{\frac{5}{3}}$$\;\eta_{W}^{\varphi }$ Where$\;K_{L}$$,A_{r}$$,\;\eta_{A_{r}}$$,\eta_{w}$ and *φ* are geometry dependent	Only tested for a square planar model with very few samples	10–20
3D Finite Element Simulators [31,32,33]	ANSYS Maxwell, COMSOL Multiphysics, etc.	Computationally intensive Long run times Need to implement the inductor design each time	Low
This study	$L_{ellipse}=\frac{µN^{2}d_{avg\_ellipse}C_{1}}{2}\left(\ln\left(C_{2}/\tau_{ellipse}\right)+C_{3}\tau_{ellipse}+C_{4}\tau_{ellipse}{}^{2}\right)$ Here $d_{avg\_ellipse}=\frac{\left(d_{in\_c}+d_{out\_c}\right)}{2}$$,\;\tau_{ellipse}=\frac{\left(d_{out\_c}–\;d_{in\_c}\right)}{\left(d_{out\_c}+d_{in\_c}\right)}$$,\;\mathrm{and}\;C_{i}$ is geometry dependent	Less accurate when the inductance is <1 μH	5 *

* Error reported here is the absolute % error between the numerical model and experimental results.

## Data Availability

All the data is already presented in the form of tables and figures.

## References

[1] Teaw E., Hou G., Gouzman M., Tang K.W., Kesluk A., Kane M., Farrell J.A. Wireless Health Monitoring System. Proceedings of the 2005 IEEE Conference on Information Acquisition.

[2] Yazdandoost K.Y., Kohno R. Wireless Communications for Body Implanted Medical Device. Proceedings of the 2007 Asia-Pacific Microwave Conference.

[3] Ammarullah M.I., Santoso G., Sugiharto S., Supriyono T., Kurdi O., Tauviqirrahman M., Winarni T.I., Jamari J. (2022). Tresca stress study of CoCrMo-on-CoCrMo bearings based on body mass index using 2D computational model. J. Tribol..

[4] Nelson B.D., Karipott S.S., Wang Y., Ong K.G. (2020). Wireless Technologies for Implantable Devices. Sensors.

[5] Molosky V. (2019). The Influence of Identifiable Personality Traits on Nurses’ Intention to Use Wireless Implantable Medical Devices. Ph.D. Thesis.

[6] Kanaan A.I., Sabaawi A.M. (2021). Implantable Wireless Systems: A Review of Potentials and Challenges. Antenna Syst..

[7] Soliman M., Chowdhury M., Khandakar A., Islam M., Qiblawey Y., Musharavati F., Nezhad E.Z. (2021). Review on Medical Implantable Antenna Technology and Imminent Research Challenges. Sensors.

[8] Farooq M., Amin B., Kraśny M.J., Elahi A., Rehman M.R.U., Wijns W., Shahzad A. (2022). An Ex Vivo Study of Wireless Linkage Distance between Implantable LC Resonance Sensor and External Readout Coil. Sensors.

[9] Lu D., Yan Y., Deng Y., Yang Q., Zhao J., Seo M., Bai W., MacEwan M.R., Huang Y., Ray W.Z. (2020). Bioresorbable Wireless Sensors as Temporary Implants for In Vivo Measurements of Pressure. Adv. Funct. Mater..

[10] Park J., Kim J.-K., Patil S.J., Park J.-K., Park S., Lee D.-W. (2016). A Wireless Pressure Sensor Integrated with a Biodegradable Polymer Stent for Biomedical Applications. Sensors.

[11] Takahata K., DeHennis A., Wise K., Gianchandani Y. A wireless microsensor for monitoring flow and pressure in a blood vessel utilizing a dual-inductor antenna stent and two pressure sensors. Proceedings of the 17th IEEE International Conference on Micro Electro Mechanical Systems. Maastricht MEMS 2004 Technical Digest.

[12] Lu D., Yan Y., Avila R., Kandela I., Stepien I., Seo M., Bai W., Yang Q., Li C., Haney C.R. (2020). Bioresorbable, wireless, passive sensors as temporary implants for monitoring regional body temper-ature. Adv. Healthc. Mater..

[13] Chen L.Y., Tee C.K., Chortos A., Schwartz G., Tse V., Lipomi D.J., Wong H.S.P., McConnell M., Bao Z. (2014). Continuous wireless pressure monitoring and mapping with ultra-small passive sensors for health monitoring and critical care. Nat. Commun..

[14] Raju S., Wu R., Chan M., Yue C.P. (2013). Modeling of Mutual Coupling Between Planar Inductors in Wireless Power Applications. IEEE Trans. Power Electron..

[15] Schormans M., Valente V., Demosthenous A. (2018). Practical Inductive Link Design for Biomedical Wireless Power Transfer: A Tutorial. IEEE Trans. Biomed. Circuits Syst..

[16] Wang F., Zhang X., Shokoueinejad M., Iskandar B.J., Webster J.G., Medow J.E. (2018). Spiral planar coil design for the intracranial pressure sensor. Med. Devices Sens..

[17] Charkhabi S., Jackson K.J., Beierle A.M., Carr A.R., Zellner E.M., Reuel N.F. (2020). Monitoring Wound Health through Bandages with Passive LC Resonant Sensors. ACS Sens..

[18] Ong K., Grimes C., Robbins C., Singh R. (2001). Design and application of a wireless, passive, resonant-circuit environmental monitoring sensor. Sens. Actuators A Phys..

[19] Grimes C.A., Mungle C.S., Zeng K., Jain M., Dreschel W.R., Paulose M., Ong K.G. (2002). Wireless Magnetoelastic Resonance Sensors: A Critical Review. Sensors.

[20] Farooq M., Iqbal T., Vazquez P., Farid N., Thampi S., Wijns W., Shahzad A. (2020). Thin-film flexible wireless pressure sensor for continuous pressure monitoring in medical applications. Sensors.

[21] Ammouri A., Belloumi H., Salah T.B., Kourda F. (2014). Experimental analysis of planar spiral inductors. Proceedings of the 2014 International Conference on Electrical Sciences and Technologies in Maghreb (CISTEM).

[22] Ahn C., Allen M. (1998). Micromachined planar inductors on silicon wafers for MEMS applications. IEEE Trans. Ind. Electron..

[23] Mohan S.S., Hershenson M.d., Boyd S.P., Lee T.H. (1999). Simple accurate expressions for planar spiral in-ductances. IEEE J. Solid-State Circuits.

[24] Mutashar S., Hannan M.A., Samad S.A., Hussain A. (2014). Analysis and Optimization of Spiral Circular Inductive Coupling Link for Bio-Implanted Applications on Air and within Human Tissue. Sensors.

[25] Deng W.-J., Wang L.-F., Dong L., Huang Q.-A. (2018). LC Wireless Sensitive Pressure Sensors With Microstructured PDMS Dielectric Layers for Wound Monitoring. IEEE Sensors J..

[26] Chen P.-J., Saati S., Varma R., Humayun M.S., Tai Y.-C. (2010). Wireless Intraocular Pressure Sensing Using Microfabricated Minimally Invasive Flexible-Coiled LC Sensor Implant. J. Microelectromechanical Syst..

[27] Greenhouse H. (1974). Design of Planar Rectangular Microelectronic Inductors. IEEE Trans. Parts, Hybrids, Packag..

[28] Eroglu A. (2011). Planar Inductor Design for High Power Applications. Prog. Electromagn. Res. B.

[29] Yue C., Ryu C., Lau J., Lee T., Wong S. (1996). A physical model for planar spiral inductors on silicon. Int. Electron Devices Meeting. Tech. Dig..

[30] Hurley W., Duffy M. (1997). Calculation of self- and mutual impedances in planar sandwich inductors. IEEE Trans. Magn..

[31] Aldoumani M., Yuce B., Zhu D. (2021). Using the Variable Geometry in a Planar Inductor for an Optimised Performance. Electronics.

[32] Hussain I., Woo D.-K. (2021). Self-Inductance Calculation of the Archimedean Spiral Coil. Energies.

[33] Yin Y., Liu Z., Zheng J., Chen L., Wu S., Wang S., Yan Z., Pan X. (2019). The Effects of Position on the Wear Debris Detection with Planar Inductor. Sensors.

[34] Derkaoui M., Benhadda Y., Hamid A. (2020). Modeling and simulation of an integrated octagonal planar transformer for RF systems. SN Appl. Sci..

[35] Gibbs R., Moreton G., Meydan T., Williams P. (2018). Comparison between Modelled and Measured Magnetic Field Scans of Different Planar Coil Topologies for Stress Sensor Applications. Sensors.

[36] Grover F.W. (1946). Inductance Calculations: Working Formulas and Tables.

[37] Huei S.G., Esa M., Kordesh A.V. (2003). RF spiral planar inductor designs-preliminary results. Fac. Electr. Eng. Univ. Technol. Malays..

[38] Crols J., Kinget P., Craninckx J., Steyaert M. An analytical model of planar inductors on lowly doped silicon substrates for high frequency analog design up to 3 GHz. Proceedings of the 1996 Symposium on VLSI Circuits. Digest of Technical Papers.

[39] Dill H.G. (1964). Designing inductors for thin film applications. Electron. Des..

[40] Wheeler H. (1928). Simple Inductance Formulas for Radio Coils. Proc. IRE.

[41] Salnikov A.S., Goryainov A.E., Dobush I.M., Kalentyev A.A., Garays D.V. Approach to scalable modeling for planar inductor using EM simulation and a few samples measurement. 2017 IEEE MTT-S International Conference on Numerical Electromagnetic and Multiphysics Modeling and Optimization for RF, Microwave, and Terahertz Applications (NEMO).

[42] Taylor L., Margueron X., Le Menach Y., Le Moigne P. (2017). Numerical modelling of PCB planar inductors: Impact of 3D modelling on high-frequency copper loss evaluation. IET Power Electron..

[43] Pacurar C., Topa V., Racasan A., Munteanu C. Inductance calculation and layout optimization for planar spiral inductors. Proceedings of the 2012 13th International Conference on Optimization of Electrical and Electronic Equipment (OPTIM).

[44] Zheng C., Li W., Li A.-L., Zhan Z., Wang L.-Y., Sun D.-H. (2016). Design and Manufacturing of a Passive Pressure Sensor Based on LC Resonance. Micromachines.

[45] Liang Y., Ma M., Zhang F., Liu F., Liu Z., Wang D., Li Y. (2019). An LC Wireless Microfluidic Sensor Based on Low Temperature Co-Fired Ceramic (LTCC) Technology. Sensors.

[46] Melati R., Hamid A., Thierry L., Derkaoui M. (2013). Design of a new electrical model of a ferromagnetic planar inductor for its integration in a micro-converter. Math. Comput. Model..

[47] Hildebrand F.B. (1976). Advanced Calculus for Applications.

[48] Abderahim A., Koularambaye M., Chatelon J.P., Capraro S., Piétroy D., Rousseau J.J. (2020). A method to determine winding losses in integrated inductors and separate skin and proximity effects. SN Appl. Sci..

[49] Kuhn W., Ibrahim N. (2001). Analysis of current crowding effects in multiturn spiral inductors. IEEE Trans. Microw. Theory Tech..

[50] Ammouri A., Ben Salah T., Morel H. (2017). A spiral planar inductor: An experimentally verified physically based model for frequency and time domains. Int. J. Numer. Model. Electron. Networks, Devices Fields.

[51] Islam A.B., Islam S.K., Tulip F.S. (2013). Design and Optimization of Printed Circuit Board Inductors for Wireless Power Transfer System. Circuits Syst..

[52] DeBoi B.T. (2019). On the Accuracy of Impedance Measurements and the Influence of Fixturing.

